# A rapid and versatile combined DNA/RNA extraction protocol and its application to the analysis of a novel DNA marker set polymorphic between *Arabidopsis thaliana *ecotypes Col-0 and Landsberg *erecta*

**DOI:** 10.1186/1746-4811-1-4

**Published:** 2005-08-23

**Authors:** Kenneth Berendzen, Iain Searle, Dean Ravenscroft, Csaba Koncz, Alfred Batschauer, George Coupland, Imre E Somssich, Bekir Ülker

**Affiliations:** 1 Max-Planck-Institute for Plant Breeding Research, Department of Developmental Biology, Carl-von-Linné Weg 10, D-50829 Köln, Germany; 2 Philipps-Universität, Biology-Plant Physiology/Photobiology, Karl-von-Frisch-Str. 8, D-35032 Marburg, Germany; 3 Max-Planck-Institute for Plant Breeding Research, Department of Plant-Microbe Interactions, Carl-von-Linné Weg 10, D-50829 Köln, Germany

## Abstract

**Background:**

Many established PCR-based approaches in plant molecular biology rely on lengthy and expensive methods for isolation of nucleic acids. Although several rapid DNA isolation protocols are available, they have not been tested for simultaneous RNA isolation for RT-PCR applications. In addition, traditional map-based cloning technologies often use ill-proportioned marker regions even when working with the model plant *Arabidopsis thaliana*, where the availability of the full genome sequence can now be exploited for the creation of a high-density marker systems.

**Results:**

We designed a high-density polymorphic marker set between two frequently used ecotypes. This new polymorphic marker set allows size separation of PCR products on agarose gels and provides an initial resolution of 10 cM in linkage mapping experiments, facilitated by a rapid plant nucleic acid extraction protocol using minimal amounts of *A. thaliana *tissue. Using this extraction protocol, we have also characterized segregating T-DNA insertion mutations. In addition, we have shown that our rapid nucleic acid extraction protocol can also be used for monitoring transcript levels by RT-PCR amplification. Finally we have demonstrated that our nucleic acid isolation method is also suitable for other plant species, such as tobacco and barley.

**Conclusion:**

To facilitate high-throughput linkage mapping and other genomic applications, our nucleic acid isolation protocol yields sufficient quality of DNA and RNA templates for PCR and RT-PCR reactions, respectively. This new technique requires considerably less time compared to other purification methods, and in combination with a new polymorphic PCR marker set dramatically reduces the workload required for linkage mapping of mutations in *A. thaliana *utilizing crosses between Col-0 and Landsberg *erecta *(L*er*) ecotypes.

## Background

PCR and RT-PCR (reverse transcriptase-PCR) are the most widely used analytical methods in plant genetics and molecular biology, providing simple tools for studying the segregation of mutations and monitoring the transcription of wild type and mutant alleles of genes in different plant tissues. Like several other classical methods (e.g. Southern and northern hybridization analysis of nucleic acids), PCR and RT-PCR applications also require a sufficient amount and quality of nucleic acids suitable for these assays. Because most well-established protocols include procedures based on the use of either potentially toxic chemicals or expensive commercial kits, numerous quick DNA isolation methods have been developed to promote large-scale genomic applications during the past years [1]. One of the major disadvantages of these quick isolation methods is that they are not suitable for applications requiring amplification of DNA fragments greater than 2 kb in size. Additionally, the available DNA purification methods have not been combined with rapid isolation of RNA from plant tissue.

Upon completion of the *A. thaliana *genome sequence, a major goal of post-genomic research is to understand the function and regulation of over 26000 genes in this model species. Several EMS (ethylmethansulfonate) and T-DNA mutagenized populations offer valuable genetic resources for wide-scale functional genomics studies in *A. thaliana *[2-6]. These studies require high-throughput DNA and RNA isolation from tens to thousands of plants. Progress in the molecular and genetic characterization of EMS and T-DNA insertion mutants is thus largely dependent on the speed, simplicity and quality of nucleic acid isolation methods. Map-based cloning of mutant alleles generated by EMS or radiation mutagenesis has been simplified by developing various pooling strategies, which are aided by well-characterized molecular markers. Many linkage mapping techniques are based either on enzymatic digestion of PCR products [7], or on the use of SNaP shot assays (SNP polymorphism markers; [8]), which may require separation on large, labor intensive acrylamide gels and detection by silver staining or radiography. Linkage mapping strategies in *A. thaliana *are still restricted by the number of known polymorphisms available between various ecotypes, such as Col-0 and L*er*. The frequent use of segregating populations derived from Col-0 × L*er *crosses, especially in the study of flowering time and plant development, would thus significantly benefit from a larger set of well-characterized and tested markers. Therefore, to develop a facile map based cloning approach, we refined the current design of polymorphic markers such that all polymorphic markers can now be resolved on 3% (w/v) agarose gels and detected by ethidium bromide staining.

To facilitate high-throughput application of our new polymorphic markers, as well as PCR-based identification and characterization of insertion mutants, we have developed a simple technique for rapid nucleic acid isolation from minimal quantity of *A. thaliana *tissue. This technique isolates both DNA and RNA templates, the quantity and quality of which are sufficient for PCR and RT-PCR analyses, respectively. Our data illustrate that this new technique can considerably accelerate PCR screening for T-DNA knockout mutations and greatly facilitates tracking segregating progeny of Col-0 × L*er *crosses, which we used for identification of an EMS-induced mutation affecting the regulation of flowering time in *A. thaliana*.

## Results

### The Sucrose Prep method for rapid isolation of nucleic acid templates for PCR analysis

We have systematically tested various protocols optimized for DNA isolation at room temperature for the efficiency of PCR amplification while omitting the inhibitory component EDTA. Combinations of DNA isolation protocols from Edwards *et al*. [9] and Walbot and Warren [10] were thus compared using ~2.5 mg (~3 mm^2^) of *A. thaliana *leaf tissue in 50 μl of extraction mixture, from which 1 μl was used as template in a routine PCR application. We have found that one of the variant extraction buffers, hereafter called Sucrose Solution, exhibited no change in the efficiency of PCR amplification judged by ethidium bromide-stained agarose gels in response to varying the pH from 7.0 to 8.0, or changing the salt and sucrose concentrations between 200 to 400 mM NaCl and 300 to 440 mM sucrose, respectively (data not shown). Due to the presence of sucrose in the extraction buffer, we named this nucleic acid extraction method the Sucrose Prep. In agreement with Thomson and Henry [11], we found that heating the crude extract for 10 min followed by centrifugation for 5 sec at 6000 g eliminated nearly all debris that interferes with PCR amplification.

**Figure 1:**
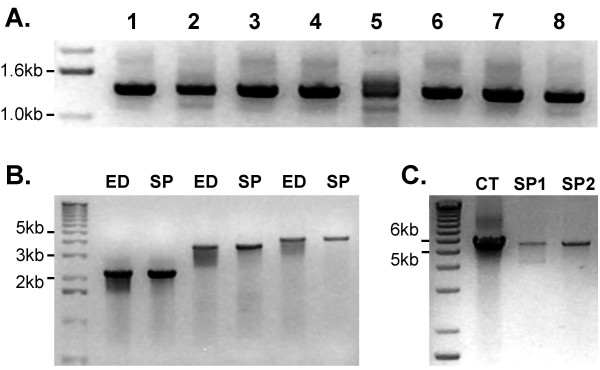
**Efficiency of PCR amplification using DNA templates prepared by the Sucrose Prep**. (A) Extracts were prepared from various tissues using 10 mg sample/200 μl Sucrose Solution. (1) 4 week-old leaf, (2) senescent leaf, (3) anthers, (4) petals, (5) sepals, (6) gynoecium, (7) petiole, (8) 8–12 days old embryos. (B) Comparison of DNA templates prepared by the methods of Edwards (ED; Edwards *et al*., [9]) and Sucrose Prep (SP) in long-range PCR amplification. Optimized PCR conditions were used to amplify 2.2, 3.7, and 4 kb size products. (C) Long-range PCR amplification on DNA template isolated by CTAB (CT; see methods) and Sucrose Prep using LA TaKaRa polymerase. Sucrose Prep replicates show the inhibition of PCR amplification by inclusion of plant debris in the reaction (SP1) in comparison to samples avoiding contamination with debris (SP2).

The Sucrose Prep protocol was further optimized by controlling sampling size. Optimal results were obtained by harvesting of leaf tissue with a 500 μl Eppendorf cap punch (yielding about 10 mg fresh tissue) in 200 μl of Sucrose Solution. Multiple samples were stored on ice prior to extraction and were ground using either sterile pipette tips or plastic pestles. The extracts could be stored at 4°C overnight or at -20°C for longer term storage, but were normally used immediately. We could store samples at -20°C for up to 4 weeks with no detectable decrease in PCR amplification efficiency. Following long term storage however, samples required re-heating before PCR amplification. Successful PCR amplification was obtained with *A. thaliana *DNA samples extracted from 2 to 4 week-old leaf material, petals, sepals, stigmas, styles, anthers, and embryos. The maximum size of products obtained in routine PCR amplification reactions was around 3 kb. Using optimized thermal cycling conditions, no difference was observed for products up to 4 kb when compared to DNA templates prepared according to Edwards *et al*. [9]. By limiting the amount of plant debris carried over into the mixture, PCR product sizes of up to 5.5 kb were obtained (Figure 1).

### Novel marker set for mapping between *A. thaliana *ecotypes Landsberg *erecta *and Col-0

**Figure 2:**
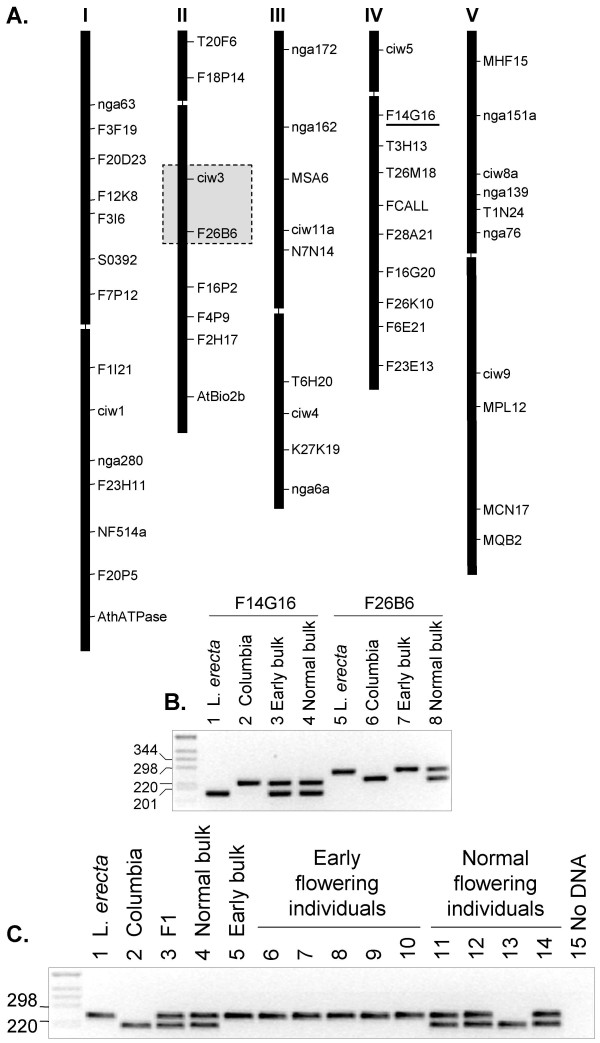
**Chromosomal location of polymorphic markers and their use for genotyping**. (A) Chromosomal location of the 51 polymorphic Col-0/L*er *markers is depicted graphically and the sequence of each primer pair and fragment polymorphism is listed in Table 1. The region on chromosome II that is linked to the early flowering phenotype is boxed. (B) PCR amplification of DNA markers F14G16 on chromosome IV and F26B6 on chromosome II from the parents and early and normal flowering F_2 _DNA bulks. For DNA marker F14G16 both the L*er *and Col-0 alleles were amplified from the early flowering bulk demonstrating that the marker is not linked to the early flowering mutation. Only the L*er *allele of DNA marker F26B6 was amplified from the early flowering DNA bulk indicating the marker is linked to the early flowering mutation. (C) PCR amplification of DNA marker F26B6 from the parents and progeny.

The availability of the entire genome sequence of *A. thaliana *ecotype Col-0 and the high abundance of genomic sequence from Landsberg *erecta *(L*er*) allowed us to design a new set of markers that show polymorphism between these ecotypes [12,13]. A novel set of 51 DNA markers were identified at regular intervals across all five chromosomes (Table 1; Figure 2A). This marker set allows mapping at a 10 cM (~2 Mbp) resolution.

**Table 1:** List of chromosomal positions of BSA markers detecting polymorphism between *A. thaliana *ecotypes Col-0 and L*er*.

**Chr**	**Marker**	**Forward primer (5'-3')**	**Reverse primer (5'-3')**	**Col-0 (bp)**	**L*er *(bp)**
I	nga63	GCCTAAACCAAGGCACAGAAG	TCATCAGTATTCGACCCAAG	87	99
I	F3F19	CCACAAAACAATTTGGTTCACTC	TCCCGTTGGGGATATTAAAG	100, 143	243
I	F20D23	TTATGCCAACTCATGTGGAAAG	TGTCAAAGCGTCTGGTTCTG	233	254
I	F12K8	ACCAACACCACAACAAACGAC	CTTTTTCTGTTCTTCCGCTATTC	171	192
I	F3I6	AGATGGAAGAGGAGGAGATGG	TGCATGTATATGATGAGCGAGAG	251	311
I	SO392	GTTGTTGATCGCAGCTTGATAAG	TTTGGAGTTAGACACGGATCTG	142	156
I	F7P12	TCGAGGATATGTTTCGTGTTTG	ACAGTTTTGATGCATTGTGTGAG	315	100, 215
I	F1I21	TCGTAAATTGTGACTGGGAGA	CCCTGTAGATCTGTTGTTTTAG	308	113, 195
I	ciw1	ACATTTTCTCAATCCTTACTC	GAGAGCTTCTTTATTTGTGAT	159	135
I	nga280	CCTGATCTCACGGACAATAGTG	GGCTCCATAAAAAGTGCACC	106	86
I	F23H11	GATATGGGAGTAAGTATGAAATCGG	TTCGTCCGGGTAAAAGTCAAG	300	250
I	NF514a	GTTGAGTCTTGGCATCACAGTTC	CTGCCTGAAATTGTCGAAAC	221	240
I	F20P5	GATACGTTCAAAATTAGGGACTTC	TGTATTTTGCTAATTGAGGTTATGG	218	186
I	AthATPase	CCTGGGAACGGTTCGATTCGAG	GTTCACAGAGAGACTCATAAACCA	86	70
II	T20F6	CGTTCGAAACTGAATTAGCTG	ACCATCTTTGTTGAGCCCTTC	297	347
II	F18P14	ATTCCCGCAATTTATTTTGTTC	GTTTGATGGCAGATTTGTTTTC	123	144
II	ciw3	GGAAACTCAATGAAATCCACTT	GTGAACTTGTTGTGAGCTTTGA	230	200
II	F26B6	CTCTATCTGCCCACGAACAAG	GCCATTGCAAAAGAACATCAG	233	274
II	F16P2	CAGCAATCAAATAACGTGGTG	CTCTCTTCTTTCTTCGCCATTAG	237	167
II	F4P9	TGGTCCATACCCATTTCATAAC	ATGAATTTTCATTCTACTGTTTTG	299	262
II	T2H17	ATTGCATACCACGCAGTTCAC	CCATTTTGCCCTTTCCTTCTAC	250	274
II	AthBIO2b	TGACCTCCTCTTCCATGGAG	TTAACAGAAACCCAAAGCTTTC	141	209
III	nga172	AGCTGCTTCCTTATAGCGTCC	CCATCCGAATGCCATTGTTC	162	136
III	nga162	CATGCAATTTGCATCTGAGG	CTCTGTCACTCTTTTCCTCTGG	107	89
III	MSA6	TTGGAGGTGCTCTTAGGTTC	GGGCTTTTCACATACGCTTTC	175	225
III	ciw11a	GTTTTTTCTAATCCCCGAGTTGAG	GAAGAAATTCCTAAAGCATTC	192	242
III	N7N14	CAATACACTTTATCCAGATGCTG	GGGATTTGTTGATTGAAAAAGGAC	150	143
III	T6H20	CGGCTGAAACTTGGAAGGGAC	AGGAAGAACGTGTGATTGTG	273	293
III	ciw4	GTTCATTAAACTTGCGTGTGT	TACGGTCAGATTGAGTGATTC	189	215
III	K27K19	TGCTTTTGAAGAGATGGTTATTAGG	CCCCATTTCACTTATCATTGG	216	198
III	nga6a	AGCGAATCCGAAAATAATGGAG	TGGATTTCTTCCTCTCTTCAC	159	137
IV	ciw5	GGTTAAAAATTAGGGTTACGA	AGATTTACGTGGAAGCAAT	164	144
IV	F14G16	ACAAACCGATCAGCATTCAAG	GCCTTTGTCACGGATTCAAC	250	198
IV	T3H13	TTTGGTGGGTCAAGAGTCAAG	GCAAAAGTCATTACGGACAATAC	275	229
IV	T26M18	CAATTAGCGGAGGCCACTTC	GGGCAAAAGCTTCCAGTAC	330	271
IV	FCALL	CCACCGTCAACATCCCTAAC	GCTCTTATACTTCTCAGCTCTTGTC	170	180
IV	F28A21	GCATCATCATTCATCACCAAC	TGTGAAGTGTTTGTCTTTGTG	198	169
IV	F16G20	TGTCAACCAATCGCCTTAGTC	TTAATGTCCATTATTGGAACGC	113	79
IV	F26K10	AGAGAGCACGATGCCTGATAG	AATGCTTCAGCGATTGAGAAC	180	205
IV	F6E21	TTCTTTGTTCAAGTTCCATGTCTC	CGGTGATTGTCTCAAGTGTTTG	199	225
IV	F23E13	TGACCGTTGAAAGTGTTGTTG	GCCCGAGAAGCCTGATAG	264	246
V	MHF15	CTCCTCCTTTAATTTTCTCTCTGTG	AGTTCCAGCTTTGGACTTCTTC	295	268
V	nga151a	ATCTCATACTGACCCATATGTTCC	ATTGTACAGTCTAAAAGCGAGAG	198	170
V	ciw8a	TACTAGTGAAACCTTTCTCAG	TTTTATGTTTTCTTCAATCAGTTAG	100	135
V	nga139	AGGGTTTCGTTTCACTATCCAG	TGAGAGCTACCAGATCCGATG	174	132
V	T1N24	CCGATGGCATAACAAGTAGAG	GGGAAAGGTACACATATACAAAAGG	383	356
V	nga76	GGAGAAAATGTCACTCTCCAC	AGGCATGGGAGACATTTACG	231	250
V	ciw9	CAGACGTATCAAATGACAAATG	GACTACTGCTCAAACTATTCGG	165	145
V	MPL12	GTCCCCAAAACCAATCATAAG	TCCGAGTGAGAAGAGAGTTTG	319	293
V	K9P8	TTATGGGTTTCTCAGAGTTTCTCAC	TTGTATGCGTTTGCTTTTTCC	284	251
V	MNC17	GTACCGGATCTGTGTTGTGAAG	GTGCTCAAGGAAATGGGATAG	168	187
V	MQB2	CTTTGATAGTAACCTTTTTCAAACCA	TGCCATTTATTTGGTCAACAC	252	231

DNA oligonucleotide primer pairs are listed according to their positions from the top to the bottom of each chromosome. The size (bp) of PCR amplification products from Col-0 and L*er *are depicted in the last two columns to the right. F, forward primer and R, reverse primer.

### Using the new marker set to map a novel early flowering mutant of L*er*

To test the efficiency of linkage mapping with the new polymorphic marker set, a L*er *line carrying T-DNA constructs overexpressing *CONSTANS *(*CO*) and *FLOWERING LOCUS C *(*FLC*), as well as a fusion between the promoter of *SUPRESSOR of OVEREXPRESSION OF CONSTANS 1 *(*SOC1*) and a beta-glucuronidase (*GUS*) gene (*35S::CO 35S::FLC 1 kb::SOC1:GUS*; [14]), was subjected to EMS mutagenesis in order to screen for mutations affecting flowering time. An early flowering mutant displaying an elongated hypocotyl when grown in white light was identified in the M_2 _generation. After demonstrating that the early flowering mutant phenotype was stably inherited to the M_4 _generation, the mutant was back-crossed with the progenitor transgenic L*er *parent. F_1 _progeny showed wild type flowering time (hereafter referred as normal flowering time), indicating that the mutation was recessive. Recessive inheritance was confirmed by the analysis of 96 F_2 _progeny, of which about one quarter showed early flowering. Subsequently, a Col-0 mapping parent was generated by crossing the *35S::CO/35S::FLC/1 kb::SOC1:GUS *transgenes from the L*er *progenitor to Col-0 four times. The early flowering L*er *mutant line was subsequently crossed with the Col-0 mapping parent, and a segregating F_2 _population was generated to map the mutation. Six early flowering plants were chosen from the F_2 _generation, and one leaf from each plant was bulked together and DNA purified using the protocol of Edwards *et al*. [9]. Similarly 6 plants showing a normal flowering phenotype were identified, their leaves were also bulked together and DNA purified.

DNA fragments were amplified from each of the DNA bulks by PCR using the entire new set of 51 poylmorphic markers. Two markers, ciw3 and F26B6 on chromosome II were identified to be linked to the early flowering mutation as both markers were homozygous for the L*er *allele from the early flowering bulk and heterozygous for the normal flowering bulk. The other DNA markers were heterozygous in both bulks indicating that they were not linked to the early flowering mutation, with the exception of three markers that most likely were linked to the loci of the T-DNA carrying the *CO*, *FLC *and *SOC1 *gene constructs (data not shown). Figure 2B shows the linked marker F26B6 and an unlinked marker F14G16 amplified from the Col-0 and L*er *parents and the DNA bulks of early and normal flowering F_2 _progeny. DNA marker F26B6 was confirmed to be linked to the mutation by PCR amplification of the marker from five early flowering and four normal flowering plants from the F_2 _mapping population (Figure 2C).

We then used the Sucrose Prep to rapidly screen 96 early flowering plants from the F_2 _population, confirming that the mutation was located between the markers ciw3 and F26B6 (data not shown). Analysis of the annotated DNA sequence for candidate genes within this region revealed *PHYB *as one of the most likely candidates responsible for the early flowering mutant phenotype. Previously, a *phyB *mutant has been demonstrated to flower earlier under long day conditions and has an elongated hypocotyl under white light conditions [15,16]. Therefore, we sequenced the *PHYB *gene from wild type L*er *and our early flowering mutant. A base substitution of cytosine to thymine was detected at position 1660 bp downstream of the *PHYB *translational start site, resulting in a premature stop codon in the first exon. This nonsense mutation is predicted to result in a truncated protein that is unlikely to be functional as it contains neither the PAS repeat domain nor the histidine kinase related domain essential for the known function of the protein.

### The Sucrose Prep as a method for identification of T-DNA insertion mutations

**Figure 3:**
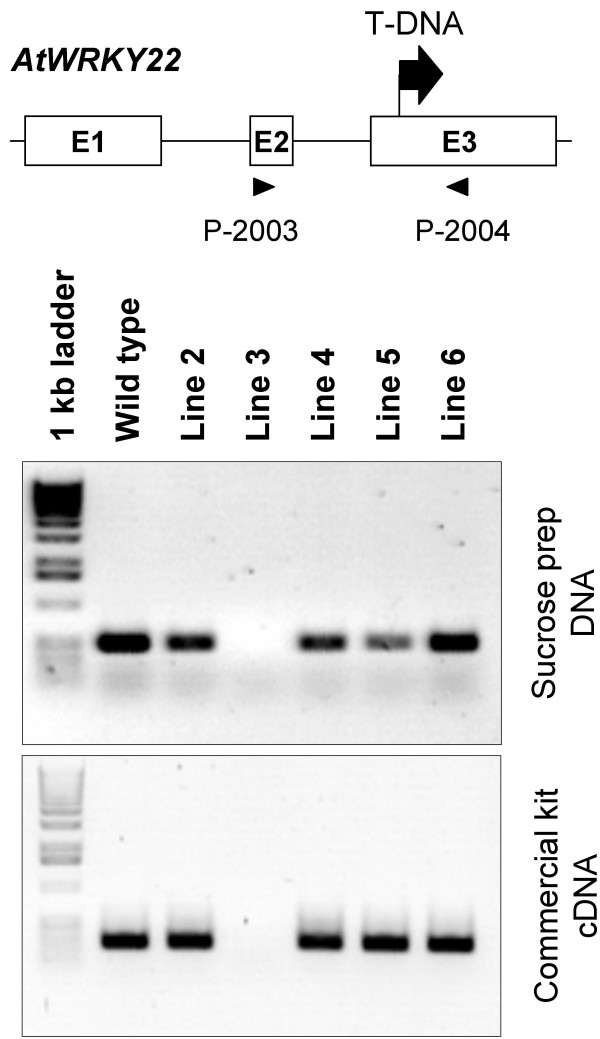
**Use of the Sucrose Prep to identify T-DNA insertion knock-out mutations**. Putative mutant plants homozygous for the T-DNA insertion were identified with the Sucrose Prep using gene specific primers for *AtWRKY22 *(Table 2) and amplification of DNA by PCR. The lines were also tested by RT-PCR for the loss of *AtWRKY22 *transcript using a commercial RNA isolation kit (Qiagen). Positions of the primers used for amplification are indicated below the schematic diagram.

To determine if the Sucrose Prep method is suitable for screening of homozygous T-DNA mutants, we screened segregating T_2 _progeny from the SALK_098205 line, in which a T-DNA was inserted in exon 3 of the *AtWRKY22 *gene (At4g01250; Figure 3). In parallel, we isolated RNA from the same plants using a commercial kit. As illustrated in Figure 3, the Sucrose Prep (DNA; upper panel) produced results that were consistent with the observed expression of the gene (cDNA; lower panel) thereby identifying line 3 as a homozygous loss-of-function mutant of *AtWRKY22*.

**Figure 4:**
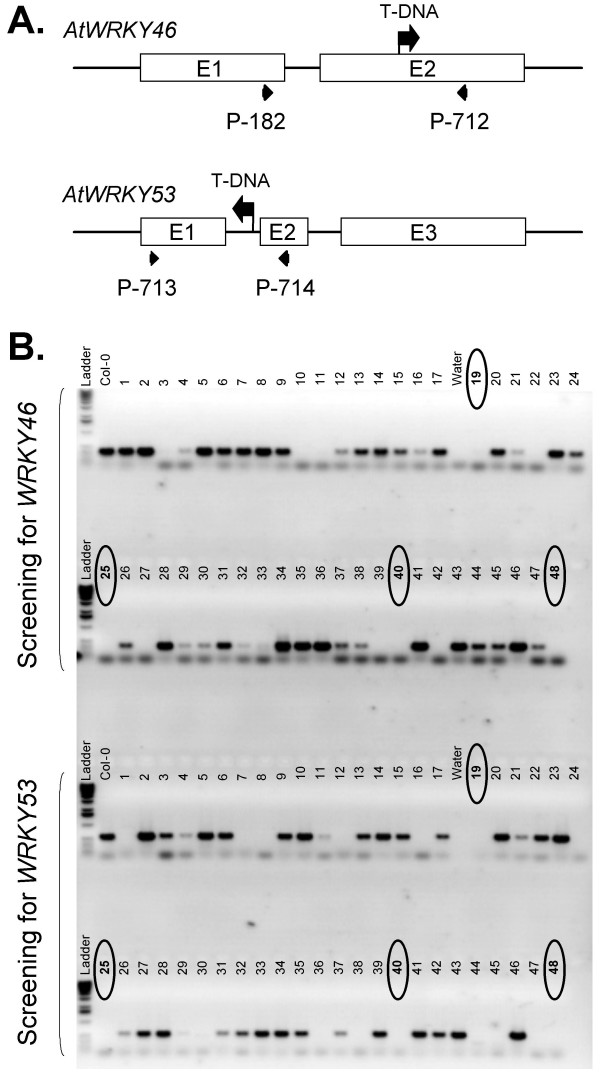
**Isolation of homozygous T-DNA insertion mutant lines carrying the *atwrky46*, *atwrky53 *double mutation**. (A) Schematic structure of *atwrky46 *and *atwrky53 *mutant alleles carrying T-DNA insertions. (B) PCR amplification using gene specific primers shown in panel A. Putative lines homozygous for the *atwrky46*, *atwrky53 *double knockout mutations fail to amplify the wild-type allele (marked by circles).

The simplicity of the Sucrose Prep also facilitated the screening for T-DNA insertions in larger populations. For example, the identification of double knockouts carrying T-DNA insertions with identical selectable markers is readily feasible using the Sucrose Prep procedure. To illustrate this point, we performed an experiment to create an *atwrky46 *(At2g46400), *atwrky53 *(At4g23810) double mutant. These WRKY transcription factors belong to the same sub-group (group III) and show very similar transcription induction kinetics in response to pathogen and elicitor treatments (data not shown). After crossing the T-DNA insertion lines (Figure 4), F_2 _progeny were screened by PCR for the loss of gene specific products. Seven candidates were immediately identified as potentially being homozygous for both insertion mutations within 90 plants assayed (Figure 4B). Two of these 7 candidates were subsequently confirmed to be true double knockouts (not shown).

### The Sucrose Prep can also effectively be used to detect the presence or absence of specific transcripts by RT-PCR

**Figure 5:**
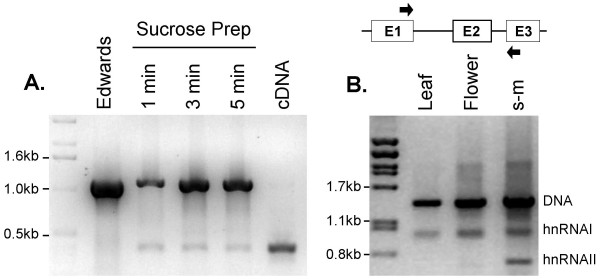
**RT-PCR analysis of RNA templates isolated by the Sucrose Prep**. (A) Amplification of a specific *SOC1 *cDNA product from leaf tissue. Leaf material was harvested and frozen in liquid nitrogen then ground in Sucrose Solution and subjected to heating at 99°C for 1, 3 or 5 min. A control PCR was performed with DNA isolated according to Edwards *et al*. [9]. A second control was performed with cDNA made from fresh leaf material prepared from RNA isolated by extraction using a commercial RNA isolation kit (Qiagen). (B) Isolation of RNA was performed as in (A) and heat treated at 99°C for 5 min. The samples were subjected to RT-PCR analysis using *AGC1-10 *specific primers, which flank the two introns depicted in the schematic diagram (expected sizes, genomic: 1300 bp, Intron I: 520 bp; Intron II: 215 bp). The cDNA product from the mature mRNA (600 bp) was detectable in shoot meristem tissues (s-m) and weakly in floral tissue, whereas only the first splicing product was observed in leaf tissues due to variations in the RNA yield (i.e., indicated by the amount of DNA product in the reactions).

To demonstrate that the Sucrose Prep method is also suitable for facile detection of specific transcripts in small amounts of plant tissue, we tested the expression of *SOC1 *(At2g45660) and *AGC1-10 *(At2g26700) genes in leaf samples by RT-PCR amplification of RNA templates prepared by the Sucrose Prep. Since RNA might be destroyed during heating, the extracts from leaf samples were subjected to various heating times of 1 to 5 min prior to RT-PCR amplification. The length of the heating step did not appear to influence the efficiency of RT-PCR amplification since the *SOC1 *transcript was detected in all samples (Figure 5). Although the amplification of the cDNA product was weaker in comparison to the efficiency obtained with the commercial RNA purification kit, the cDNA product was specific and its amplification was reproducible.

**Figure 6:**
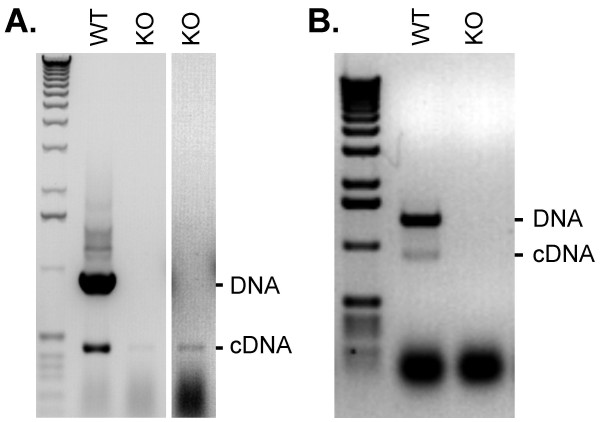
**Transcript analysis in *A. thaliana *knockout mutants**. (A) Leaf material was harvested from wild type or homozygous *atwrky36 *knockout mutant plants and the Sucrose Prep was used for RT-PCR analysis with gene specific primers. The T-DNA is located in the first intron of *AtWRKY36 *and was occasionally transcribed and spliced out since a cDNA product corresponding to a segment of mature RNA is detected in the knockout mutant. The second column is digitally enhanced to highlight the cDNA product detected in the knockout line. (B) Leaf material from wild-type and a homozygous *atwrky70 *mutant were sampled using the 'Touch-and-Go' method (see text for details). The T-DNA insertion in the gene prevented PCR amplification of both DNA and cDNA products (larger than 4 kb).

Next, we tested homozygous T-DNA knockout lines for loss of *WRKY *transcripts. A T-DNA in the *AtWRKY36 *(At1g69810) gene was localized within the first intron. This insertion resulted in a loss of detectable transcript by RT-PCR analysis compared to wild type (data not shown). RT-PCR analysis of wild-type and homozygous *atwrky36 *knockout mutant plants was performed by using the Sucrose Prep. Since the T-DNA was located in the first intron, no amplification of cDNA product was expected in the knockout mutant (Figure 6, panel A). Nonetheless, a faint cDNA product was detected by the RT-PCR assay suggesting that the T-DNA insertion was spliced out from a small fraction of primary transcripts.

### The 'Touch-and-Go' approach for PCR and RT-PCR applications

We also developed an alternative method for isolating nucleic acids from very small sample sizes designated 'Touch-and-Go'. This method eliminates the preparation steps required by the Sucrose Prep, since the extraction of DNA/RNA templates is made simply by capturing plant tissue with a pipette tip which is immediately available for the amplification of nucleic acids by PCR and RT-PCR methods respectively. In practice, leaf tissue was punctured using an RNAse-free 20 μl pipette-tip against a firm surface, i.e. a finger covered with a latex glove, and then the pipette-tip was immediately touched into 50 μl PCR solution mix in prepared reaction wells. Due to the very small amount of leaf material taken by pipette tip puncturing, the number of PCR cycles should be 35 to 40 when using this method. For plant material located in the greenhouse or the field, 10 μl water was first delivered to PCR tubes or plates kept on ice, and 'Touch-and-Go' sampling was performed by touching the water in the tubes or wells with the pipette tip containing the plant tissue. After sampling, 40 μl PCR solution mix was added to the tubes or wells in the laboratory and DNA/RNA was amplified by PCR for 40 cycles using a thermocycler. Figure 6, panel B illustrates that the 'Touch-and-Go' method can be used to PCR amplify DNA products a maximum of 1.5 kb in size. This mini-preparation method was also tested in combination with RT-PCR by monitoring for the loss of detectable expression of *AtWRKY70 *(At3g56400) in a T-DNA insertion mutant line. The *atwrky70 *insertion mutant carries the T-DNA insertion in the last exon of the gene. Figure 6, panel B illustrates that no specific cDNA signal was detectable in the homozygous *atwrky70 *knockout line in comparison to wild type extracts from which both DNA and cDNA products were well amplified. To verify that the observed lower band of the correct predicted cDNA size of is indeed *AtWRKY70*, this fragment was gel isolated and subsequently sequenced. The sequencing data clearly confirmed that this fragment represents the full-length *AtWRKY70 *cDNA fragment and that the two known introns were spliced out.

**Figure 7:**
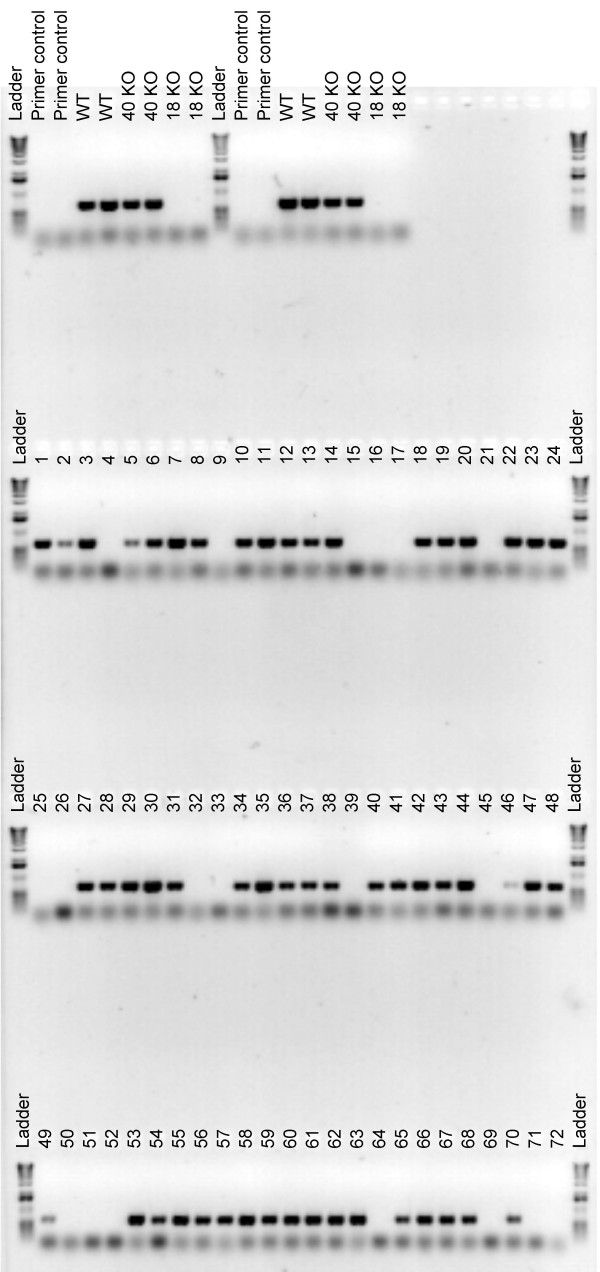
**Screening for homozygous *atwrky18 *T-DNA knockouts using the 'Touch-and-Go' method**. Seventy-two F_2 _progeny obtained by crossing of homozygous *atwrky40 *and *atwrky18 *mutants, were screened with primers flanking the T-DNA insertion site in the *AtWRKY18 *gene allowing detection of the wild-type *AtWRKY18 *allele. Amplification of a 644 bp size fragment indicates that F_2 _progeny are either wild type or heterozygous for *AtWRKY18*. No amplification suggests that the progeny are homozygous for the *atwrky18 *mutant allele. Controls, including DNA from the homozygous parental lines (knockout; KO), were replicated twice in pairs, giving a total of four independent control reactions. Primer control, lanes that contain no DNA template.

The 'Touch-and-Go' extraction method was also tested in screening for an *atwrky40*, *atwrky18 *double mutant. Figure 7 shows a screening of F_2 _progeny homozygous for the *atwrky18 *T-DNA insertion mutation. Whereas a DNA fragment of 644 bp specific for the *AtWRKY18 *locus was amplified from extracts prepared from wild type Col-0 and homozygous *atwrky40 *mutant plants, this was not the case when the assay was applied to extracts that were prepared from homozygous *atwrky18 *lines. By screening 72 segregating F_2 _progeny from an *atwrky40 *× *atwrky18 *cross, we identified 19 individuals to be homozygous for the *atwrky18 *mutation (as judged by the absence of the 644 bp PCR product). These results are in agreement with the expected Mendelian segregation ratio (i.e. 18/72; Figure 7)

### The Sucrose Prep and the 'Touch-and-Go' methods can successfully be used in other plants

**Figure 8:**
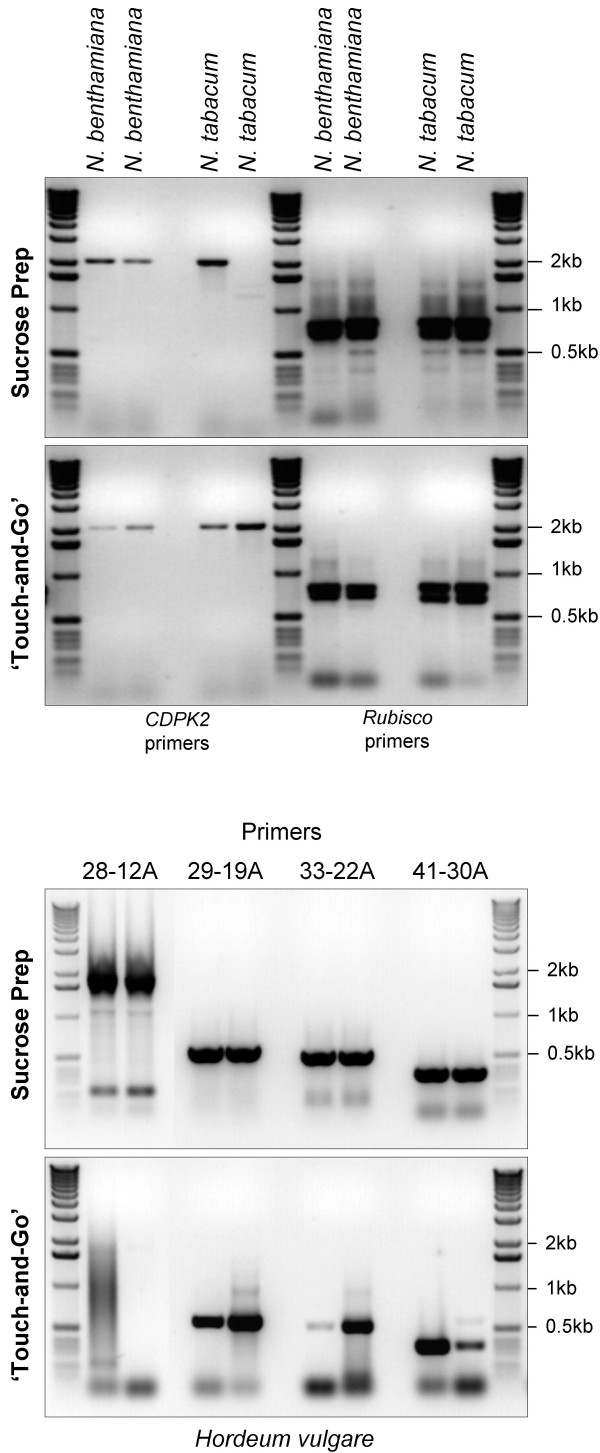
**Sucrose Prep and the 'Touch-and-Go' methods works well in other plant species**. DNA was isolated in duplicate using the Sucrose Prep or the 'Touch-and-Go' methods (indicated on the left) from leaves of one month old *N. tabacum *and *N. benthamiana *plants (upper two panels) or from leaves of 10 day-old barley plants (lower two panels). PCR was carried out for 40 cycles in a 50 μl reaction volume using primers for *NtCDPK2 *and *NtRBCS *in tobacco, and four different pairs of primers for barley. Only 10 μl of the final reaction was resolved by agarose (1%) gel electrophoresis for analysis.

We isolated DNA from the dicotyledonous crop species tobacco (*Nicotiana tabacum*) and its close relative *N. benthamiana*, as well as from the monocotyledonous crop species barley (*Hordeum vulgare*) using Sucrose Prep and the 'Touch-and-Go' methods to demonstrate their applicability for plants other than *A. thaliana*. As illustrated in Figure 8 (upper two panels), both methods produced sufficient quality and quantity of DNA that can be amplified using primers for two tobacco genes *NtCDPK2 *(calcium dependent protein kinase2) and *NtRBCS *that yield 2 kb and 0.8 kb PCR amplified DNA products respectively. Amplification failed in only one out of eight reactions using the Sucrose Prep while none failed using the 'Touch-and-Go' method. A similar isolation and PCR amplification was performed with barley tissue using four barley specific primer pairs producing varying sizes of amplified DNA fragments (Figure 8; lower two panels and Table 2). The sizes and patterns of the observed amplified DNA fragments are identical to those observed with other conventional DNA isolation methods (personal communication T. Zhao, M. Böhmer, and G. Freymark, MPIZ Köln). All of the primer pairs produced the expected size fragments (1.7, 0.7, 0.6 and 0.4 kb) in PCR analysis using DNA isolated by the Sucrose Prep. Using the Touch-and-Go' method, three primer pairs successfully amplified the expected smaller size fragments (0.7, 0.6 and 0.4 kb) but failed to amplify the largest size fragment of 1.7 kb.

**Table 2:** List of primers and T-DNA lines used

**Species/gene**	**T-DNA knockout**	**Primer No**	**Primer (5'-3')**
*AtSOC1*			GGATCGAGTCAGCACCAAACC
			CTTGAAGAACAAGGTAACCCA
			
*AtAGC1-10*			CGTTTCACTATCTCCTCCACAAG
			GGTGCTTTCAGAATGTTTACTAACGT
			
*AtWRKY22*	SALK_098205	2003	AAGAAAGTGTGCCATGTAGCAG
		2004	CCGGAGACGATGAATAAGTAGC
			
*AtWRKY46*	GABI-Kat 038C07	182	ATGGAGGAGGTTCTAGCGAGAGTC
		712	AAACGTCTTTACCATCATCAAGC
			
*AtWRKY53*	SALK_034157	713	ACGAATTGGAACTAGGGAAAGAG
		714	CCATCATCAATAGAGCCATTTTC
			
*AtWRKY36*	GABI-Kat 258B10	72	CCTGCCTACAAAGATCATCTAGTTTCG
		136	ATGATCAAAGAGGAGACCGTTTC
			
*AtWRKY70*	GABI-Kat 752F08		ATGGATACTAATAAAGCAAAAAAGC
			AGATAGATTCGAACATGAACTGAAG
			
*AtWRKY18*	GABI-Kat 328G03		CATGGGTTCATTTCAAATTTTCG
			CGATCTGCTCATGTTGCTGATGATG
			
*NtCDPK2*			ATGGGCAACGCATGCGGCGG
			GATGACTCTCAAAGCCATTTTC
			
*NtRBCS*			CCTCTGCAGCAGTTGCCACC
			CCTGTGGGTATGCCTTCTTC
			
*Hordeum vulgare*		28-12A	ATACCTGCACAGCCACAAGTC
			GCAACTTCGCCTCTACGTTC
			
*Hordeum vulgare*		29-19A	ACATGTGAGCTTGCTGGTTG
			TGGGGGATGGTTAATGGTAG
			
*Hordeum vulgare*		33-22A	CCTGCCGATGTAATCTGGTT
			GATCTTTGCCATGTCTGTTTCG
			
*Hordeum vulgare*		41-30A	AACATGCAAGCACACGTCAT
			CATGATTGCTGTGGCTGACT

**Figure 9:**
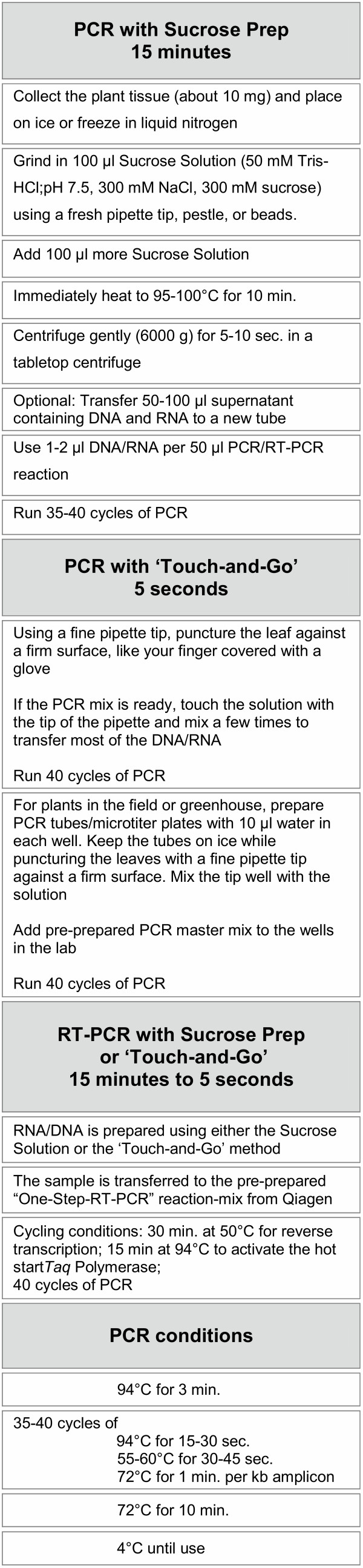
Flow diagram of the Sucrose Prep and the 'Touch-and-Go' methods for PCR and RT-PCR applications.

Flow diagrams for the Sucrose Prep and the 'Touch-and-Go' methods for PCR and RT-PCR applications are shown in Figure 9.

## Discussion

The Sucrose Prep is not unique in being a quick DNA isolation protocol. Kasajima *et al*. [18] have exploited the method of Edwards *et al*. [9] to develop a rapid method for marker and transgene detection, and have demonstrated amplification of fragments up to 1.4 kb in size. As Langridge *et al*. [19] and Petersen *et al*. [20], we have also observed that DNA can be extracted by grinding the plant tissue in pure water and transferring a sample aliquot of the extract to a PCR reaction or that DNA templates can be delivered by adding a small amount of tissue to a PCR reaction mixture (data not shown). Therefore, we tried using extremely small amounts of tissue sampled with pipette tips by puncturing leaf tissue and immediately touching the tips into prepared PCR reaction mixtures. This 'Touch-and-Go' method is comparable in PCR amplification efficiency of DNA fragments up to about 1 kb with the Sucrose Prep and other rapid nucleic acid isolation methods. The stability of the Sucrose Prep in providing templates for numerous PCR or RT-PCR reactions however encouraged us to continue optimization of the method in connection with various high-throughput applications.

Alkaline lysis with NaOH [21-23] has also been successfully used in rapid isolation of DNA, however PCR amplification of DNA fragments are typically smaller than 2 kb in size. A common step between the majority of rapid procedures and the Sucrose Prep is the inclusion of a 'boiling' step. Burr *et al*. [24] used a thermal cycling protocol from 65°C to 96°C for a total time of 11.5 min, whereas Thomas and Henry [11] had optimized their protocol for DNA extraction from dried tissue by heating for 10 min at 95°C. Many quick DNA preparation methods dilute out contaminants from the harvested tissue, which interfere with the PCR reaction, by raising the extraction volume [22-26]. Sucrose Prep also employs such a dilution step by using about 50 μl extraction buffer for 2.5 mg tissue. Another component of the Sucrose Solution is the use of high salt, which is also employed in the protocol of Wang *et al*. [22] and is a principal component of DNA extraction buffers described by Edwards *et al*. [9] and Walbot and Warren [10].

The Sucrose Prep has been optimized for *A. thaliana *tissue, but is also suitable for DNA isolation from other species, including tobacco and barley (Figure 8). Therefore, Sucrose Prep should also work for species such as maize, wheat, rice, potato, tomato and other plants that have low to moderate concentrations of phenolics and starch

The combination of bulk segregant analysis with our new polymorphic DNA marker set proved very effective in rapidly locating a mutation of interest within a 10 cM interval. Tracking F_2 _segregation with the Sucrose Prep dramatically eased the analysis, as we employed a DNA shaker and thermal cycling blocks for heating, making future optimisation with robots possible. DNA sequencing of a candidate gene identified a mutation in the first exon of the *PHYB *gene, causing a premature stop codon at amino acid 554. The resulting truncated protein is predicted not to contain the PAS domain, which are important in phytochrome function and mediate interaction with putative signalling partners [27-29].

The Sucrose Prep also proved useful in screening for homozygous T-DNA insertion mutants and it was particularly useful for rapid identification of double homozygous knock-out lines that carried T-DNA insertions bearing identical selectable markers (Figure 3 and Figure 5). Occasionally, certain PCR primers did behave differently between conventional DNA preparations and templates obtained by the Sucrose Prep (Figure 1 and Figure 4). Nonetheless, in most cases where a primer combination did not work with Sucrose Prep, they also failed to produce PCR amplification with conventional methods under the aforementioned size limits (data not shown).

Pre-screening of segregating F_2 _or T_2 _EMS- and T-DNA-induced mutation populations with the Sucrose Prep greatly reduced the number of lines requiring further characterization. Upon fast screening with Sucrose Prep, detailed analysis always led to the identification of homozygous mutant lines that were confirmed by other DNA and RNA isolation methods.

RNA isolation from plants is often a lengthy process requiring toxic chemicals or expensive kits, and requires a very clean practice due to vast contamination of RNAses. A quick RNA isolation method is therefore highly desirable. We have demonstrated above that plant extracts prepared by the Sucrose Prep are also suitable for RT-PCR assays (Figures 5 and 6). Due to high concentration of DNA in the extracts, the primers must be designed in exons separated by introns in order to distinguish DNA from cDNA (Figure 6). DNA contamination is not a unique problem to our approach but is common to several other RNA isolation methods. The only major disadvantage of our quick RNA isolation method is that the RNA to DNA ratio is very low as compared to other methods where RNA is concentrated through several steps. However, our method is very useful and even superior to other methods in certain applications requiring speed and use of limited amounts of plant tissue. Thus, the method is particularly useful if cells expressing the gene of interest are restricted to certain tissue, such as hydathodes, major and minor veins, emerging young leaves, flower organs such as nectaries, flower abscission zones, sepals petals, anthers, gynoecium, root tips, as well as local pathogen infected tissues and islands of cells generated by transposon jumping. One of the limitations of our method is that it is not suitable for genes that are expressed at very low levels. The 'Touch-and-Go' method is not suitable for quantitative RT-PCR applications, due to the varying amount of RNA isolated during sampling.

Using the Sucrose Prep and the 'Touch-and-Go' methods, we have identified homozygous T-DNA knockouts for the *AtWRKY36 *and *AtWRKY70 *genes (Figure 6). With respect to PCR amplification of DNA samples, we found the 'Touch-and-Go' method extremely useful and time saving, especially when screening for the presence or absence of PCR products of less than 500 bp in size. We had however variable success rates if the size of the expected PCR products was larger than 1 kb. As with the Sucrose Prep, the 'Touch-and-Go' method worked very well for tobacco in amplifying DNA fragments up to 2 kb, however, in barley it failed to amplify the largest predicted fragment of 1.7 kb and the amount of amplification product varied for fragments less than 1 kb in length. This difference observed in the reproducibility of DNA isolation between tobacco and barley is possibly due to the lower number of cells that are disrupted in barley plants upon puncturing with the pipette tip. Nevertheless, the simplicity, speed and reproducibility of the 'Touch-and-Go' approach and the robustness and relative speed of the Sucrose Prep method makes them ideal for high-throughput PCR based screens in alternative transgenic approaches replacing the use antibiotic resistance selectable markers [31].

## Conclusion

In comparison to other rapid nucleic acid isolation protocols described for plant samples, the Sucrose Prep is thus far the only extraction protocol, which is shown to be compatible with simultaneous isolation of DNA and RNA templates. This minimal-step nucleic acid isolation method can be combined with the use of our high resolution marker set that can be resolved on agarose gel after amplification with PCR to perform fast and precise mapping of mutations using DNA polymorphisms between *A. thaliana *ecotypes Col-0 and L*er*. We anticipate that the utilization of Sucrose Prep as well the 'Touch-and-Go' method will facilitate the improvement of automated high-throughput genomic techniques used in functional genomics studies of the model plant *A. thaliana*, as well as in other plants species, including important crops.

## Methods

### The Sucrose Prep

Sucrose Solution: 50 mM Tris-HCl pH 7.5, 300 mM NaCl and 300 mM sucrose.

(A) Individual samples: approximately 10 mg of leaf tissue was placed directly in 200 μl Sucrose Solution and ground at room temperature or on ice using a pipette tip or pestle. The samples were then heated to 99–100°C for 10 min and then briefly spun at 2000–6000 g for 5 sec. The samples were placed on ice until PCR. One μl of the supernatant was used for PCR, avoiding debris.

(B) Multiple sample 96-well format: between 10–20 mg leaf tissue from 14 d-old plants was harvested as leaf discs into 96 well plates. Metal balls (3 mm, tungsten carbide beads) were added and shaken in a Retsch MM300 shaker for 10 sec, then 300 μl of Sucrose Solution was added, the plate heated at 99°C for 10 min and placed on ice until use. Following storage at 4°C or -20°C, samples were reheated at 99–100°C for 10 min and then immediately placed on ice.

### The 'Touch-and-Go' method

Leaves were punctured against a firm surface, like a finger covered with a latex glove using a fine pipette tip (TipOne from Starlab GMBH, catalog no. S1111-3000 or S1110-3000). The DNA/RNA on the tip of the pipette was transferred to the pre-prepared PCR solution in the PCR tubes by touching the tip of the pipette to the solution and pipetting up and down a few times. For plants in the field or greenhouse, 10 μl water was aliquoted into PCR tubes or microtiter plates. The tubes/plates were kept on ice while puncturing the leaves with a fine pipette tip against a firm surface and DNA/RNA the tip was transferred into water by pipeting up and down. After returning the samples to the laboratory, 40 μl PCR master mix was added to each well. Thermocycling with the 'Touch-and-Go' method requires 40 cycles of PCR amplification.

### CTAB method

The CTAB protocol used was developed by Murray and Thompson [32], modified from Rogers and Bendich [33] and adapted by Rios *et al*. [2].

### Plant Growth Conditions

*A. thaliana *plants were grown under a 16 h photoperiod at 20°C in a greenhouse.

### EMS Mutagenesis

Approximately 6,500 *35S::CO 35S::FLC 1 kb:SOC1::GUS co-2 *seeds were mutagenized by imbibition in 0.3% ethyl methanesulfonate (EMS; Sigma) for 8 to 9 h, followed by washing with 0.1 M Na_2_SO_4 _and distilled water. The M_1 _mutagenized seed was planted into about 260 pools each containing 25 M_1 _seeds. About 300 M_2 _seed from each pool were sown under long day conditions and scored for flowering time.

### PCR and RT-PCR

Routine PCR: 3 min 94°C, 35–40 cycles of: (30 sec 94°C, 45 sec 55°C, 1 min 72°C), 10 min 72°C, 4°C until analysis. 2.5 μM each gene specific primers, 2.5 mM dNTPs, 5–10 U (0.5–1 μl) *Taq *polymerase (Invitrogen), 1 × *Taq *Buffer (commercially supplied). For products larger than 2 kb, 0.5 U of enzyme LA *Taq *polymerase (TaKaRa) was substituted for Invitrogen *Taq *polymerase and the PCR protocol from Rios *et al*. [2] was used.

Qiagen OneStep RT-PCR Kit (catalog no. 210210) was used following the manufacturer's recommendations. For isolation of cDNA, RNA was extracted with RNeasy Plant Mini Kit from Qiagen (catalog no. 74904) following the manufacturer's instructions.

Primers and T-DNA knockout lines are listed in Table 2.

### Gel documentation

All of the agarose gel pictures are ethidium bromide stained gels and the images are inverted in Adobe Photoshop.

## Competing interests

The author(s) declare that they have no competing interests.

## Authors' contributions

KB developed the Sucrose Prep, initiated collaboration with IS to use the Sucrose Prep for mapping purposes and together with BU was involved in the design, coordination, and drafting of the manuscript. IS designed the novel marker set between Col-0 and L*er*, IS and DR mapped the mutation in the *PHYB *gene and assisted in drafting the manuscript. BU developed the 'Touch-and-Go' method and demonstrated that the nucleic acids isolated by Sucrose Prep or the 'Touch-and-Go' methods are suitable for RT-PCR assays, tested all of these methods in screening T-DNA knockouts in Arabidopsis and demonstrated that these methods are also suitable for tobacco and barley. CK, GC and IES provided the laboratory facilities, gave valuable experimental advises and extensively helped in drafting the manuscript. AB and CK are the Ph.D supervisors of KB. All authors were involved in reading, correcting and approving the final version of the manuscript.
